# Tacrolimus treatment in women with repeated implantation failures

**DOI:** 10.1002/rmb2.12558

**Published:** 2024-01-09

**Authors:** Koji Nakagawa, Rikikazu Sugiyama

**Affiliations:** ^1^ Center for Reproductive Medicine and Implantation Research Sugiyama Clinic Shinjuku Tokyo Japan

**Keywords:** placental abruption, recurrent pregnancy loss, repeated implantation failure, tacrolimus, Th1/Th2

## Abstract

**Background:**

Tacrolimus is an immunosuppressive drug that works as a calcineurin inhibitor to improve the reproductive outcomes for women who have experienced multiple implantation failures (RIF) and show elevated type 1 helper T (Th1)/Th2 cell ratios.

**Methods:**

In the first part of this review, we indicate how we re‐evaluated the cut‐off index for selecting the participants in a tacrolimus regimen via transferred euploid blastocysts. In the second part, we cite cases where tacrolimus has improved the live birth rate for women who have experienced recurrent pregnancy losses (PRL) and we introduce the utility of tacrolimus treatment to prevent obstetrical complications.

**Main Findings:**

After reconsideration of the cut‐off index (Th1/Th2 ≥ 11.8), however, the pregnancy rates of women with tacrolimus were significantly higher than those of women without tacrolimus. The PRL women treated with tacrolimus showed significantly lower rates of biochemical pregnancy, but higher live‐birth rates compared with women who were not treated with tacrolimus. Moreover, prior severe obstetrical complications could be controlled via the administration of tacrolimus during pregnancy.

**Conclusion:**

Tacrolimus has become indispensable in the field of solid‐organ transplantation, and in the near future, it should become an essential agent in the reproductive field, as well.

## 1 INTRODUCTION

The success rate of assisted reproductive technology (ART) has dramatically improved, and there is no doubt that many women have benefitted from this treatment. Nonetheless, it is undeniable that some women continue to experience repeated implantation failures (RIFs) despite receiving several embryo or blastocyst transfers.^1^, ^2^, ^3^ The common definition of RIF refers to women who have experienced three failures of fresh or cryopreserved‐thawed embryo transfer (ET) despite the use of good‐quality embryos.^1^ It is well known that the requirements for embryo implantation include a good embryo, adequate decidualization, and appropriate maternal response, which equates to embryonic, uterine, and maternal systemic factors that include an adequate immune response. “A good embryo” refers to a blastocyst with the ability to become a baby—in other words, a euploid blastocyst. According to a recent report, at least one or two euploid blastocysts would be included among three morphologically good blastocysts derived from women between the ages of 25 and 35 years.^4^ It follows that RIF women must have received at least one or two euploid blastocysts; nevertheless, these women did not achieve pregnancy. Based on this point of view, RIF has causes other than embryonic factors, and these other causes are likely interfering with embryo implantation.^5^

Helper T (Th) cells express CD4 on their surface, and this protein plays a critical role in Th cell activation by binding to human leukocyte antigen (HLA) class II molecules, which aid the immune system in recognizing foreign substances. Th cells can be divided into two general subgroups with different characteristics and functions: Type 1 helper (Th1) and Type 2 helper (Th2) cells. These subpopulations can be differentiated by the pattern of cytokine production (chemical messengers).^6^, ^7^ Th1 cells primarily synthesize several cytokines such as interferon gamma (INF‐γ), tumor necrosis factor‐beta (TNF‐β), and interleukin‐2 (IL‐2). In contrast, Th2 cells mainly produce interleukin‐4 (IL‐4), IL‐5, IL‐6, IL‐9, IL‐10, and IL‐13. The primary role of Th1 cells is to stimulate cell‐mediated responses involving cytotoxic T cells and macrophages, while Th2 cells primarily assist in stimulating B cells to produce antibodies. Th1 and Th2 cells are the leading characters in the immunological processes that determine either rejection or tolerance.^8^

Several steps are needed for Th cell activation. The first step begins with antigen‐presenting cells such as macrophages. These cells ingest infectious agents or foreign particles, and export fragments of those, so‐called antigens, to the cell surface. A surface receptor of the Th cells binds to the human leukocyte antigen (HLA) complex. In the next step, Th cell activation occurs either through cytokines stimulation or the co‐stimulatory reactions of a signaling protein, B7. B7 is found on the surface of the antigen‐presenting cells,^9^ which binds to CD28, on the surface of the Th cell.^10^ As a result of Th cell activation, the number of Th cells that recognize antigens increases, and Th cell cytokines are produced. Th cells neither directly kill infected cells nor attack other cells (foreign cells or hatched blastocysts), as cytotoxic T cells do. Th cells activate cytotoxic T cells, help macrophages to attack infected or foreign cells, or they stimulate B cells to secrete antibodies.

The first step in achieving implantation is to establish a hatched blastocyst that will be accepted by the decidualized uterine endometrium. When maternal Th cells recognize a hatched blastocyst, rather than directly attacking it, Th cells produce various cytokines, such as INF‐γ, TNF‐α, or IL‐2. At the implantation site, maternal‐fetal immune interactions involve both inhibitions of rejection immunity and immune tolerance induction. This phenomenon has also been documented in the fields of organ transplantation and cancer cell biology.^11^, ^12^, ^13^, ^14^

It has become common knowledge that the establishment of pregnancy is an immunological condition wherein Th2 cells show superiority over Th1. By contrast, when Th1 shows superiority over Th2 the result is immunological rejection. As a consequence, Th1 superiority tends to reject transferred embryos.^15^ Based on this theory, implantation failures are thought to be a case of allograft rejection.^16^

## 2 PAST DECADE

### 2.1 The motivation for ordering tacrolimus treatment

Tacrolimus is an immunosuppressive drug that functions as a calcineurin inhibitor^17^ and is currently being used to help reduce the risk of organ rejection among organ transplant recipients.^18^ In 2011, we started to use tacrolimus as an immunosuppressive agent for infertility patients who had experienced RIFs during ART treatment. We thought that immunological reactions between the transferred embryo and women's bodies, particularly that of uterine decidual endometrium during implantation, resembled those of transplantation recipients. Therefore, we regarded the embryo as a transplanted organ, and this was why we administered the immunosuppressive drug tacrolimus to the RIF patients.^19^ We strongly believe that the embryo in the uterine endometrium of RIF patients was being rejected immunologically as a foreign body. Kwak‐Kim et al.^20^ recognized before others that the RIF women showed an increase in Th1 cytokines, and they considered immunological rejection to be one of the causes of RIF.^21^

Then, we measured type 1 helper T (Th1)/Th2 cell ratios randomly among the RIF women who experienced more than 3 in‐vitro fertilization (IVF) failures and were surprised that 37.7% of them showed elevated Th1/Th2 cell ratios.^22^ We speculated that the reason for their multiple IVF failures should be immunological rejection, and we recommended administration of the immunosuppressive agent tacrolimus for women who showed elevated Th1/Th2 cell ratio.^19^

Allogenic organ transplantation has become a common medical procedure, and the contribution of the progress of immunosuppressive agents has been effective in this field. Tacrolimus is now commonly used to achieve pregnancy, and the regimen is administered during pregnancy, which has allowed many female recipients to give birth.^23^ Tacrolimus is an immunosuppressive drug, which is currently being used to help reduce the risk of organ rejection among organ transplant recipients, and works as a calcineurin inhibitor.^24^ Tacrolimus binds with FK506 binding protein in the cytoplasm, and this complex inhibits calcineurin action. Inhibition of calcineurin action blocks the activation of the nuclear factor of activated T cell (NF‐ATC) and blocks cytokine secretion.^25^ A simple explanation is that tacrolimus blocks the communication between Th1 cells and macrophages or cytotoxic T cells (CTLs) to remove or reject the transferred embryos. Tacrolimus and Ciclosporin, are both calcineurin inhibitors, but tacrolimus more effectively reduces the incidence of acute rejection^26^ and shows a more favorable lipid profile.^27^ These findings were helpful in our decision to use tacrolimus in the reproductive field.

The mechanism of immunological rejection during implantation was complicated, but herein we will attempt to explain this mechanism in terms as simple as possible. When a blastocyst is about to be implanted into the uterine endometrium, Th1 cells can detect a blastocyst in the uterine endometrium. Th1 cells recognize a blastocyst as a non‐self‐object because a blastocyst is considered as a semi‐allograft. Consequently, Th1 cells allow the macrophages, or CTL, to recognize a non‐self‐object via secretion of various cytokines such as INF‐γ and TNF‐α. Then, the macrophages phagocyte a blastocyst. Upon administration, tacrolimus cuts off the communication between Th1 cells and macrophages. This is the mechanism that tacrolimus uses.

### 2.2 How to select the patients for tacrolimus treatment

All of the selected women with RIF had their T‐cell count measured before embryo transfer. Peripheral blood can be measured by flow cytometric analysis, and the specific staining of lymphocytes is performed. Th1 cells are defined as CD4+ lymphocytes with intracellular IFN‐γ but without IL‐4. Th2 cells are detected as CD4+ lymphocytes with intracellular IL‐4 but without IFN‐γ. The ratio of IFN‐γ to IL‐4 positive Th cells is expressed as the Th1/Th2 ratio. The cut‐off value for starting tacrolimus treatment is a Th1/Th2 cell ratio ≥ 10.3.^19^ The dose of tacrolimus is selected depending on the Th1/Th2 cell ratios.^28^ Administration of tacrolimus is begun 2 days before embryo transfer. The current values of regulatory T cell (Treg; CD4 + FOX3 + CD23+) and Th17 (CD4 + IL‐17+)/Treg ratios are also added for the evaluation in the cases of immunological rejection of RIF patients. Low Treg value (<5.0) or elevated Th17/Treg ratios (≥0.6) are indicators of the need for tacrolimus treatment (unpublished data).

Recently, an interesting paper proved our hypothesis using uterine endometrial cells.^29^ There was a concern that our criteria of immunological rejection showing elevated Th1/Th2 cell ratios were determined by peripheral blood analysis and could reflect the environment in the uterine endometrium. In that paper, the expression of several cytokines (IL‐4, IL‐10, INF‐γ, and leukemia inhibitory factor [LIF]) was evaluated in the uterine endometrial tissue of patients showing an elevated peripheral blood Th1/Th2 cell ratio (≥10.3) with or without tacrolimus treatment. From the results of that paper, the protein expression of INF‐γ and INF‐γ/IL‐4, or INF‐γ/IL‐10 showed a positive correlation.^29^ This result clarified how an immunological imbalance such as elevated Th1/Th2 cell ratios in the peripheral blood reflects a similar immunological imbalance in the uterine endometrial tissue.

We wondered if there was an appropriate peripheral blood Th1/Th2 cell range. It seemed that the very high values of a peripheral blood Th1/Th2 cell ratio were optimal neither for implantation nor for the maintenance of pregnancy. High Th1/Th2 cell ratio values were caused by either elevated Th1 cell values or a decrease in the Th2 value. Of these two conditions, we speculated that the former could greatly affect the interference of implantation or maintenance of pregnancy rather than the latter condition because the much higher inflammatory cytokines such as IFN‐γ and TNF‐α would be secreted by the increased Th 1 cells.^28^ On the other hand, a very low Th1/Th2 cell ratio value was also considered less than optimal. This condition is usually caused by an elevated Th2 cell value. The increase in Th2 cells increases allergic reactions and activates an auto‐immune response. These facts suggest that peripheral blood Th1/Th2 cell values should be in the appropriate range, and over or under values of the appropriate range are not good for embryonic implantation, maintenance of pregnancy, or allergic reactions.

### 2.3 Tacrolimus improves reproductive outcomes when using euploid blastocysts

In this review, the patients showed elevated Th1/Th2 cell ratios, which we suspected were caused by immunological rejection that led to a higher rate of RIF. Our first report concerning tacrolimus treatment indicated that the clinical pregnancy rate (CPR) in the tacrolimus treatment group was significantly higher than that in the control,^19^ and since then, via the use of tacrolimus, higher CPRs have been achieved among RIF patients who show elevated Th1/Th2 cell ratios.^28^

**FIGURE 1:**
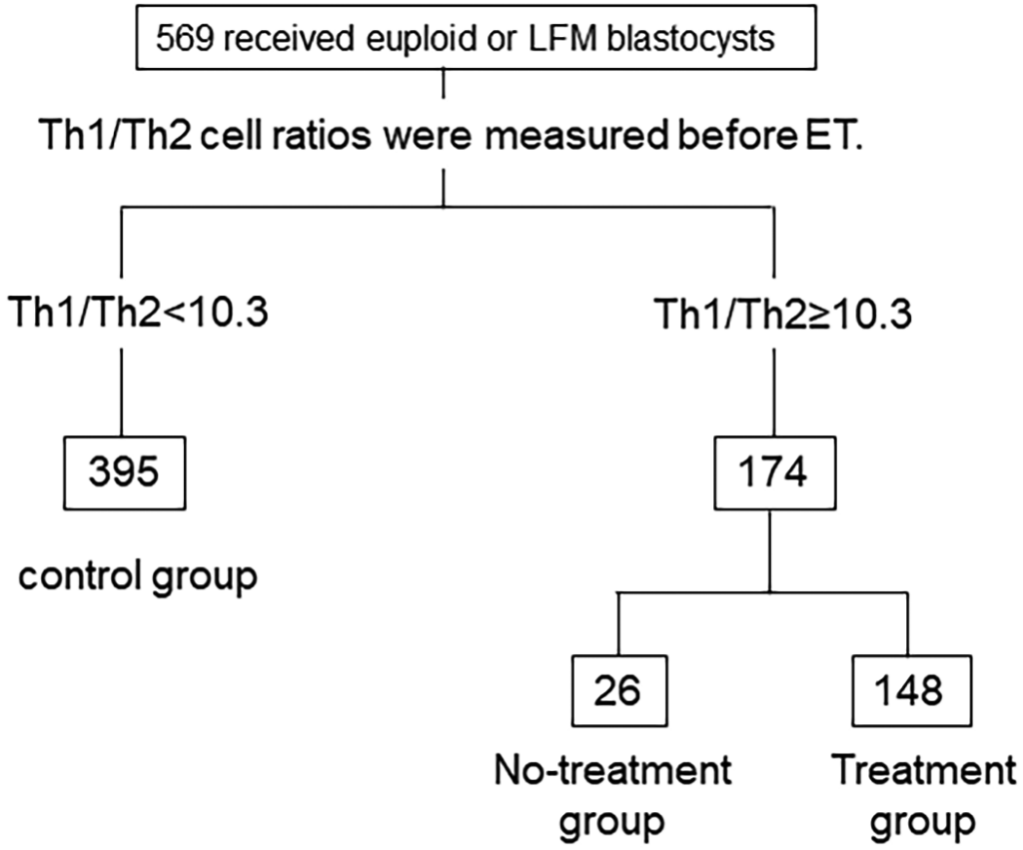
This is the flow diagram of participants who showed Th1/Th2 cell ratios equal to 10.3 or above, or not, before receiving euploid blastocyst. A total of 569 women who received euploid or low‐frequency mosaic blastocysts by preimplantation genetic testing for aneuploidy were recruited for this evaluation. Among them, 148 women received tacrolimus (as the treatment group) whereas the others received no drugs (no‐treatment group). Women showing Th1/Th2 cell ratios of 10.3 or less were considered as the control (*n* = 395). LFM, low‐frequency mosaic.

We strongly believe that tacrolimus treatment contributed to the success of our RIF patients. However, our first and second studies did not include the use of euploid blastocysts,^19^, ^28^ and embryonic concerns could not be ruled out. Therefore, we attempted to confirm the effectiveness of the tacrolimus treatment on women with RIF who showed elevated Th1/Th2 cell ratios when using euploid blastocysts. Between September 2020 and November 2021, a total of 569 women who received euploid or low‐frequency mosaic blastocysts by preimplantation genetic testing for aneuploidy (PGT‐A) were recruited for this evaluation (Figure 1). The procedures of PGT‐A were previously described.^30^ All participants were measured for Th1/Th2 cell ratios before embryo transfer (ET), and 174 women showing Th1/Th2 cell ratios of 10.3 or above were divided into two groups. Among them, 148 women received tacrolimus (as a treatment group) and the others received no drugs (no‐treatment group). The tacrolimus dose was decided according to the previous report^17^ (11). Women showing 10.3 or less of their Th1/Th2 cell ratios were considered as the **control** group (*n* = 395). The hCG positive and GS rates in the treatment group were 73.0% and 64.2%, respectively, and these two rates were comparable to those in the no‐treatment group (69.2% and 57.7%, respectively). In contrast, both rates in the control group were 70.6% and 60.0%, respectively. The miscarriage rates in the treatment and no‐treatment groups reflected no significant differences at 16.8% and 6.7%, respectively (Table 1). Based on these results, the use of Th1/Th2 cell ratios of 10.3 or above was questioned for its utility as the criteria value for tacrolimus treatment. Therefore, we decided that the criteria value for tacrolimus treatment should be reconsidered.

**TABLE 1:** The reproductive outcomes of the tacrolimus treatment for the repeated implantation failure women with transferring euploid blastocyst.

	Treatment group	No‐treatment group	Control	*p*
Th1/Th2 cell ratios ≥ 10.3				
Patients, *n*	148	26	395	—
Biochemical pregnancy, *n*	108	18	279	—
Biochemical pregnancy rate, %	73.0	69.2	70.6	n.s
Clinical pregnancy, *n*	95	15	237	—
Clinical pregnancy rate, %	64.2	57.7	60.0	n.s
Miscarriage, *n*	16	1	31	—
Clinical miscarriage rate, %	16.8	6.7	13.1	n.s
Th1/Th2 cell ratios ≥ 11.8				
Patients, *n*	112	12	445	
Clinical pregnancy, *n*	75	4	268	
Clinical pregnancy rate, %	67.0	33.3	60.2	<0.05^a^

^a^ Treatment group vs. No treatment group.

We attempted to gather volunteer women for the establishment of a new Th1/Th2 cell ratio criteria value. Volunteer women, who were 40 years old or younger with more than one healthy child, no history of infertility treatment and who had experienced no or only 1 miscarriage, were recruited for this evaluation. We found 45 women who registered Th1/Th2 cell ratios with a mean value +1SD of 11.8, which we then set as a new criteria value.^31^ We re‐analyzed the 569 women who had received a euploid transfer, and 124 of them (21.8% [124/569]) were classified into the elevated Th1/Th2 cell group (Th1/Th2 cell ratios ≥11.8). Among them, 112 women were treated with tacrolimus (**treatment group**) while the others (*n* = 12) received no drug (**no‐tacrolimus group**). The clinical pregnancy rate in the tacrolimus group was 67.0% [75/112], and this was significantly higher than that in the no‐tacrolimus group (33.3% [4/12], *p* < 0.05, Table 1). This result confirmed our decision that the Th1/th2 cell ratio criteria value for selecting patients who should be treated with tacrolimus should be set at 11.8, or above.

### 2.4 Tacrolimus concentration

**FIGURE 2:**
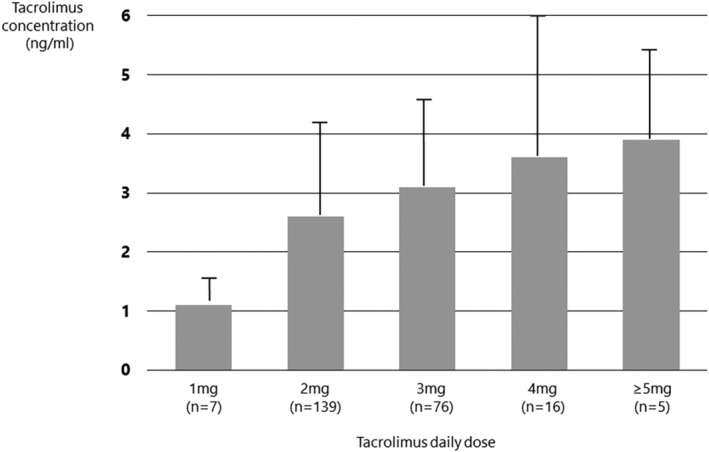
We measured 243 samples from 61 women taking between 1 and ≥5 mg of tacrolimus daily, once a day after dinner. This graph shows the concentrations of each tacrolimus daily dose. At daily dosages of 1, 2, 3, 4, and ≥5 mg, the concentrations are 1.1, 2.6, 3.1, 3.6, and 3.9 ng/mL, respectively.

The next concern was how to manage the patients who are treated with tacrolimus. Tacrolimus is an integral part of the standard immunosuppressive regimen for solid organ transplantation. The therapeutic window for tacrolimus is narrow and many factors interfere with its metabolism. Therefore, the dose of tacrolimus should reflect the peripheral blood concentration, as established in the field of solid organ transplantation.^32^ Recently, several published papers have focused on the clinical pharmacokinetics of tacrolimus.^33^, ^34^ After solid‐organ transplantation, the concentration of tacrolimus should be measured to maintain a therapeutic range. Although the use of tacrolimus for women with RIF differs from that of transplantation recipients, we have monitored the concentrations of tacrolimus since 2017. The concentration of tacrolimus determines the optimal dose in solid organ transplantation cases (5–15 ng/mL). We measured 243 samples from 61 women taking between 1 mg and ≥5 mg of tacrolimus daily, once a day after dinner. The tacrolimus concentrations of each daily dose appear in Figure 2.^22^ At a daily dosage of between 1 and ≥5 mg, the concentrations ranged from 1.1 to 3.9 ng/mL, respectively, and although these doses did not reach the optimal dose for organ transplantation, keeping the concentrations of tacrolimus within an appropriate range was justified by a higher pregnancy rate.

After the establishment of pregnancy, women continued taking tacrolimus according to the Th1/Th2 ratios and tacrolimus serum concentration. Evaluation of the Th1/Th2 cell ratios and tacrolimus concentration were performed at 12, 20, 28, and 34 weeks of gestation. When the ratios showed 10.3 or less, administration of tacrolimus ceased.

### 2.5 Safety

Tacrolimus is an immunosuppressive drug that was approved in Japan in 1993 to prevent immunological rejection for solid organ transplantation recipients. Since then, this has been spread worldwide for solid organ transplantation recipients due to few side effects by comparison with ciclosporin, which is also a calcineurin inhibitor. There were several reports of pregnant women who were solid organ transplantation recipients and who took tacrolimus before and after achieving pregnancy, and no report of adverse effects for fetus/babies was seen in these articles.^35^, ^36^ Moreover, Yocum et al.^37^, ^38^ reported that a low dose of tacrolimus (2 or 3 mg daily) did not increase the incidence of infection and they concluded that tacrolimus was safe and provided clinical benefit over a period of at least 12 months. A portion of the administration to pregnant women in the drug package of tacrolimus was changed from “not allowed” to “beneficial administration”. Based on this fact, tacrolimus was administered in a low dose (less than 4 mg daily) and determined to be a safe drug for infertile women, pregnant women, and fetuses.

## 3 NEXT DECADE

### 3.1 To prevent miscarriage

After the establishment of conception using tacrolimus, the next question was whether pregnant women should continue taking tacrolimus. The answer is Yes. The mechanism was established whereby the uterine endometrium accepts a hatched blastocyst that could be immunologically similar to the villi and could be rejected by the mother's lymphocytes. As previously established, tacrolimus has dramatically improved the implantation rate for RIF women.^28^ Based on this point of view, it is thought that tacrolimus must be effective for women who have experienced repeated pregnancy losses (RPLs) or severe obstetrical complications such as abruptio placenta or HELLP (hemolysis, elevated liver, enzyme, low platelets) syndrome.

**FIGURE 3:**
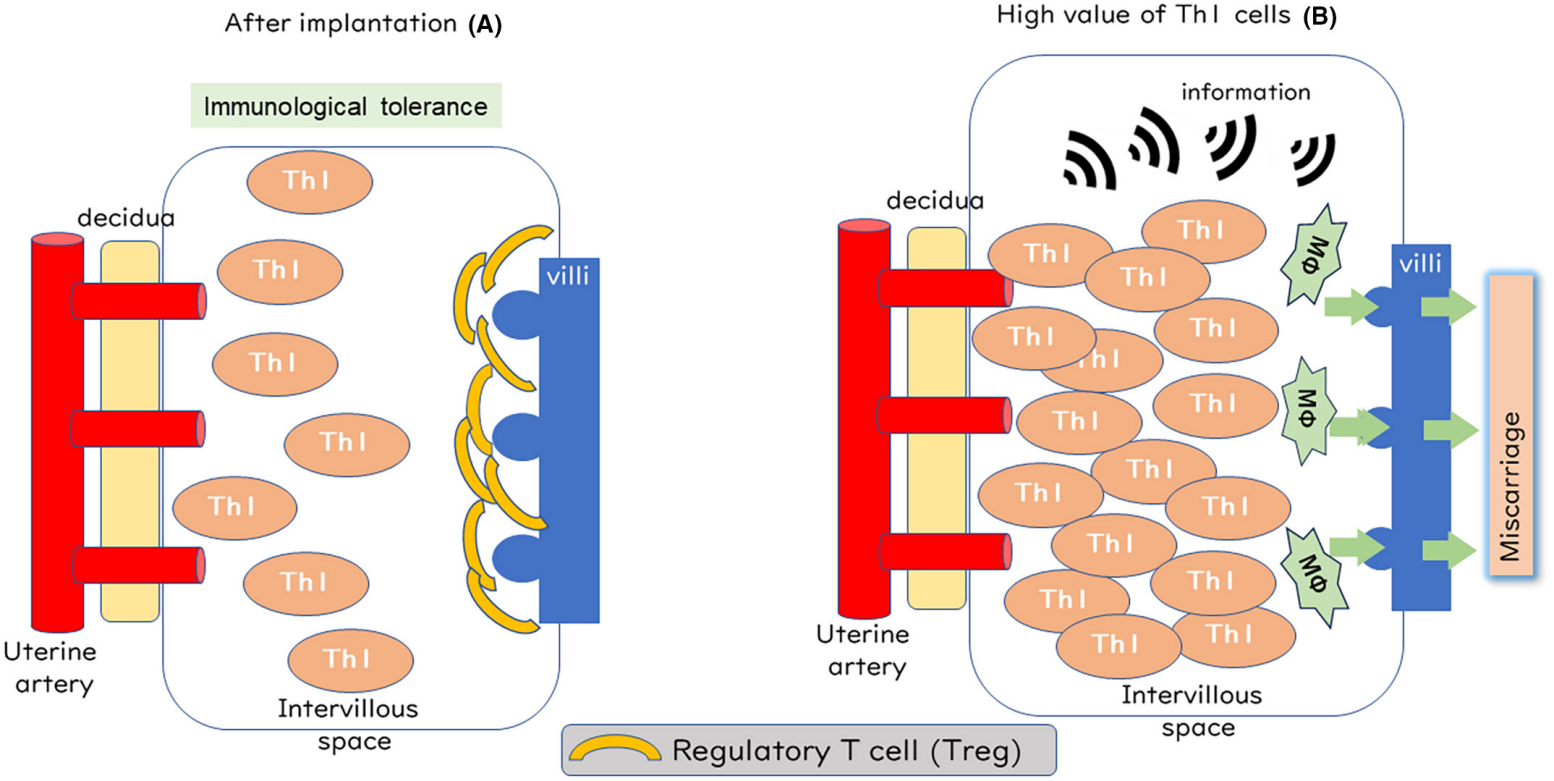
After implantation, the Th1 cells exist in the intervillous space. After the establishment of immunological tolerance, the villi should not be attacked (A). On the other hand, in the case that a high value of Th1 cells exists in the intervillous space, Th1 cells would see the villi as a non‐self object, whereby macrophages would phagocyte the blastocyst and the villi would be attacked by macrophages and cytotoxic T cells, with the consequence being a miscarriage (B).

After the establishment of pregnancy with tacrolimus treatment, we strongly recommended that the women should continue taking tacrolimus to prevent miscarriage and other obstetrical complications. After implantation, the Th1 cells exist in the intervillous space, but the villi should not be attacked by macrophages and CTLs due to an established immunological tolerance (Figure 3A). In contrast, in cases where a high number of Th1 cells exist in the intervillous space, Th1 cells easily recognize the villi as a foreign object, and then the villi would be attacked by macrophages and CTLs, which would result in a miscarriage (Figure 3B). As mentioned above, tacrolimus blocks the communication between Th1 cells and macrophages or CTLs, so the villi are not attacked or rejected. Therefore, we believed that immunosuppressive treatment with tacrolimus would improve the reproductive outcome of the women with RPLs.

About 7 years ago, we had a memorable case involving a woman who experienced 12 consecutive miscarriages and was the treated with tacrolimus (2 mg/day). This woman gave birth, although she suffered from severe hypertensive disorders with pregnancy (HDP).^39^ Three years later, this woman visited our clinic wishing to have a second baby. After confirmation of her hCG‐positive result, she started taking tacrolimus in the same dose as her more recent successful pregnancy (2 mg/day). Unfortunately, she miscarried, but a couple of months later, she became pregnant. She started taking an increased dose of tacrolimus (3 mg/day) due to her previous miscarriage, and regardless of taking 2 mg of tacrolimus, she gave birth without HDP. This particular case showed that while tacrolimus is effective for women with RPLs, immunological rejection could make them more vulnerable to obstetrical complications such as HDP.

Recently, we published a paper concerning ART outcomes for women who had experienced recurrent pregnancy losses and were treated with tacrolimus.^40^ A total of 100 pregnant women with RPLs (between 4 and 11 times) were divided into two groups, 71 were treated with tacrolimus from 1 to 4 mg daily (treatment group) and the others (*n* = 29) did not receive tacrolimus (control group). In the treatment group, the biochemical pregnancy rate was significantly lower, but the live birth rate (70.4%) was significantly higher than those in the control group (44.8%, *p* < 0.05; Table 2). Our data establish tacrolimus as a viable treatment option for women with RPLs showing immunological rejections.

**TABLE 2:** The outcomes of the tacrolimus treatment for the women with multiple pregnancy losses.

	Treatment group				Control group
	Total	Tacrolimus dose, daily			
		1 mg	2 mg	≥3 mg	
Patients, *n*	71	23	33	15	29
Biochemical pregnancy, %	4.2	8.7	3.0	0	27.6*
Clinical pregnancy, %	95.8	91.3	97.0	100	72.4**
Clinical miscarriage rate, %	29.6	47.8	27.3	6.7	55.2
Live birth rate, %	70.4	52.2	72.7	93.3	44.8**

*p < 0.05 between the total treatment group and control group; **p < 0.05, between 1 and ≥3 mg in the treatment group.

### 3.2 Use of tacrolimus to prevent severe obstetrical complications

Speaking of severe obstetrical complications such as abruptio placenta or the HELLP syndrome, the past history of the onset of placental abruption is well known to be one of the most serious risk factors (relative risk; 10–188) in the management of a subsequent pregnancy.^41^ In these cases, particular attention must be paid to managing the women who have experienced prior placental abruption once they become pregnant following tacrolimus treatment.

Currently, immune biomarkers to predict pre‐eclampsia have been reported by Gracia et al.^42^ This report showed that the decreased Treg value and elevated Th1 values were associated with the onset of preeclampsia. Based on this result, we expect tacrolimus to prevent preeclampsia for the women showing these findings. We documented 6 cases of patients who experienced obstetrical complications such as HDP, abruptio or HELLP syndrome in their prior pregnancy. These patients showed high Th1 cell values and also high Th1/Th2 cell ratios. They received 2–4 mg of tacrolimus daily for 2 days before ET and ultimately achieved pregnancy. In all cases, the administration of tacrolimus was continued during their 2nd or 3rd pregnancy until just before delivery, and all of them delivered healthy babies without severe complications (Table 3).

**TABLE 3:** Tacrolimus could manage the women who experienced obstetrical complications.

Prior obstetrical complication	Number	How to manage
HDP	1	TC only, e‐CS at 27 weeks due to acute HDP
Abruptio placenta	3	TC only, All cases, s‐CS at full term
HELLP syndrome	1	TC only, Onset of HELLP at 32 weeks
Post‐partum HELLP	1	TC only, HELLP (−)

Abbreviations: e‐CS, emergency‐cesarean section; HDP, hypertensive disorder with pregnancy; HELLP, hemolysis, elevated liver, enzyme, low platelets syndrome; s‐CS, selective‐cesarean section; TC, Tacrolimus.

Another interesting case involved an rh‐incompatible pregnancy. Although the details were described in our paper,^43^ the treatment course is briefly described here. A woman whose blood type was Rh‐negative was immunized at her first delivery (baby's blood type was Rh‐positive) and showed an elevated level of anti‐D antibody. She wished to have a second baby, but 4 ET sessions all failed. When her immunological profiles were checked, she showed high Th1/Th2 cell ratios (Th1 was 32.4, Th2 was 1.3, and ratio was 24.9). With the aid of tacrolimus, she achieved pregnancy by ET, and she continued taking tacrolimus to prevent miscarriage and an elevation of her anti‐D titer. She gave birth to a healthy baby whose blood type was Rh‐positive. We were able to manage this Rh‐incompatible pregnancy using only tacrolimus without plasmapheresis. This result was not always expected, but we speculated that tacrolimus was effective in this case because it suppressed cell‐mediated immunity, which also prevented the production of immunoglobulin due to the suppression of IL‐4 secretion.^44^ In Japan, no effective strategy appears in obstetrical textbooks for dealing with pregnant women who show a high titer of anti‐D antibodies. Based on this point of view, tacrolimus could be an effective method for treating rho‐incompatible pregnancies.

### 3.3 Safety for both mother and fetus/baby

Repeated implantation failure and PRL women with elevated Th1/Th2 ratio levels could be candidates for developing severe obstetrical complications such as preeclampsia including placental abruption. Tacrolimus inhibits calcineurin in helper T cells, and as a result, secretion of several cytokines such as IL‐2, INF‐γ, etc., are suppressed. Administration of tacrolimus for the RIF and RPL women should be continued during pregnancy because tacrolimus prevents the occurrence of obstetrical complications such as preeclampsia and placental abruption. Concerns about the safety of tacrolimus for pregnant women during pregnancy have been dispelled. Tacrolimus is classified as a class C drug by the FDA pregnancy category, and the safety of tacrolimus for both mother and fetus/baby during pregnancy has been well established in many reports of female transplant recipients who have achieved a post‐transplant pregnancy.^18^, ^45^ Moreover, we recently described how the obstetrical complications for the RIF women treated with tacrolimus were not associated with obstetrical and perinatal complications. Also, we have reported the neuromotor development of babies exposed to tacrolimus in utero and found it to be comparable to that of babies from the general population.^46^

## 4 CONCLUSIONS

**FIGURE 4:**
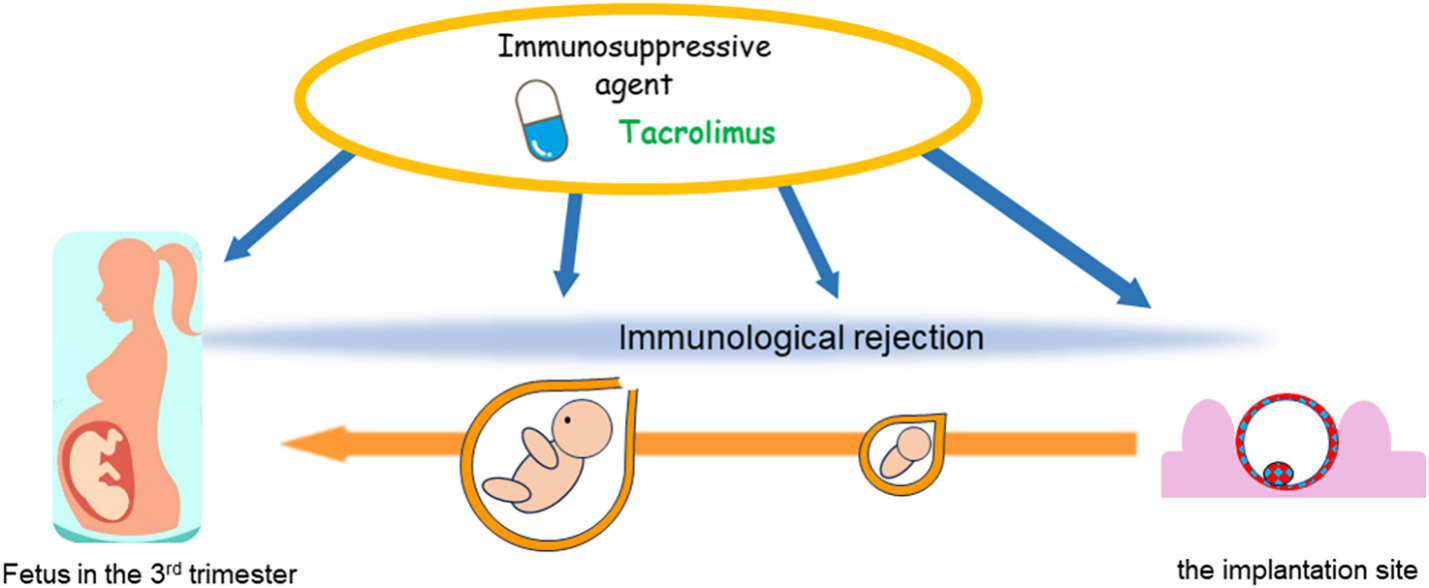
Immunological rejection might occur at any point along the continuum stretching from the moment of implantation to development of the fetus in the 3rd trimester. Immunosuppressive agent tacrolimus has demonstrated strong potential to prevent various disorders due to immunological rejection.

Immunological rejection could occur at any point along a continuum from the initiation of implantation to the development of the fetus in the 3rd trimester (Figure 4). The immunosuppressive agent tacrolimus has shown strong potential to prevent various disorders due to immunological rejection.

The benefits from tacrolimus treatment for women with both RIF and RPLs who show elevated Th1/Th2 cell ratios could even be immeasurable. Under the current health insurance system in Japan, however, tacrolimus treatment is not covered. RIF women must be treated on a self‐pay basis if they choose to use this drug for their IVF treatment, and as a consequence all procedures for IVF treatments are then not covered by insurance—the billing of mixed medical care services in the Japanese health care system is unacceptable in the current system in Japan. Thankfully, an exploratory clinical trial (jRCTs031220235) has been launched to determine the efficacy, safety and dosage of tacrolimus for women with RIF.^36^, ^47^ With the completion of this trial, tacrolimus should be approved for use by RIF women under the health insurance system in Japan.

## CONFLICT OF INTEREST STATEMENT

The authors declare no conflicts of interest. This study was conducted in accordance with the ethical standards as laid down in the 1964 Declaration of Helsinki and its later amendments or comparable ethical standards, and it was reviewed and approved by both the institutional review board of Sugiyama Clinic and the National Center for Child Health and Development. All patients received and signed an informed written consent form before entering the study, and they were also informed of the option to withdraw from the study at any time during the treatment.
